# Sufentanil Reduces Emergence Delirium in Children Undergoing Transthoracic Device Closure of VSD After Sevoflurane-Based Cardiac Anesthesia

**DOI:** 10.21470/1678-9741-2019-0334

**Published:** 2020

**Authors:** Ning Xu, Qiang Chen, Shu-Ting Huang, Kai-Peng Sun, Hua Cao

**Affiliations:** 1 Department of Cardiac Surgery, Fujian Provincial Maternity and Children’s Hospital, Affiliated Hospital of Fujian Medical University, Fuzhou, P. R. China.; 2 Department of Cardiovascular Surgery, Union Hospital, Fujian Medical University, Fuzhou, P. R. China.

**Keywords:** Emergence Delirium, Sufentanil, Dizziness, Sevoflurance, Fentanyl, Incidence, Anesthesia, Cardiac Procedures, Heart Septal Defects, Ventricular, Airway Extubation, Vertigo, Anesthesia, Intensive Care Units

## Abstract

**Objective:**

The aim of this study was to evaluate whether sufentanil can reduce emergence delirium in children undergoing transthoracic device closure of ventricular septal defect (VSD) after sevoflurane-based cardiac anesthesia.

**Methods:**

From February 2019 to May 2019, 68 children who underwent transthoracic device closure of VSD at our center were retrospectively analyzed. All patients were divided into two groups: 36 patients in group S, who were given sufentanil and sevoflurane-based cardiac anesthesia, and 32 patients in group F, who were given fentanyl and sevoflurane-based cardiac anesthesia. The following clinical data were recorded: age, sex, body weight, operation time, and bispectral index (BIS). After the children were sent to the intensive care unit (ICU), pediatric anesthesia emergence delirium (PAED) and face, legs, activity, cry, consolability (FLACC) scale scores were also assessed. The incidence of adverse reactions, such as nausea, vomiting, drowsiness and dizziness, was recorded.

**Results:**

There was no significant difference in age, sex, body weight, operation time or BIS value between the two groups. Extubation time (min), PEAD score and FLACC scale score in group S were significantly better than those in group F (*P*<0.05). No serious anesthesia or drug-related side effects occurred.

**Conclusions:**

Sufentanil can be safely used in sevoflurane-based fast-track cardiac anesthesia for transthoracic device closure of VSD in children. Compared to fentanyl, sufentanil is more effective in reducing postoperative emergence delirium, with lower analgesia scores and greater comfort.

**Table:** 

Abbreviations, acronyms & symbols	
**ASA**	**= American Society of Anesthesiologists**
**BIS**	**= Bispectral index**
**FLACC**	**= Face, legs, activity, cry, consolability**
**ICU**	**= Intensive care unit**
**PAED**	**= Pediatric anesthesia emergence delirium**
**SPSS**	**= Statistical Package for the Social Sciences**
**SQI**	**= Signal quality index**
**TEE**	**= Transesophageal echocardiography**
**TTE**	**= Transthoracic echocardiography**
**VSD**	**= Ventricular septal defect**

## INTRODUCTION

Transthoracic device closure of ventricular septal defect (VSD) in children, which combines the advantages of surgical repair and transcatheter device closure of VSD, has been widely used in the clinic in recent years, especially in China^[1,2]^. The technique has the advantages of a small incision, without the need for cardiopulmonary bypass, a simple operation process and quick recovery. Sevoflurane is often used for cardiac anesthesia in pediatric surgery. It has the advantages of rapid analepsia, little airway stimulation, and stable hemodynamics. However, the incidence of emergence delirium is as high as 40%, which affects the postoperative recovery of children, increases the difficulty of postoperative nursing and aggravates the psychological burden on children and their families^[3,4]^. Sufentanil has a strong analgesic capacity and sedative effect and lasts longer^[5]^. It has a mild effect on the cardiovascular system and no effect on histamine release, so it can effectively reduce postoperative pain in children^[6]^. Therefore, it is widely used in the induction and maintenance of anesthesia in pediatric cardiac surgery. The purpose of this study was to observe the effect of sufentanil on postoperative emergence delirium in children undergoing transthoracic device closure of VSD after sevoflurane-based fast-track cardiac anesthesia.

## METHODS

The ethics committee of our university approved the present study. All treatment measures followed the Declaration of Helsinki. In addition, all guardians of the patients were informed of the content of the study and signed the written consent form.

We retrospectively analyzed the clinical data of 68 children who underwent transthoracic device closure of VSD in our heart center between February 2019 and May 2019. According to the classification of the American Society of Anesthesiologists (ASA), all patients belonged to ASA class I-II. All children were diagnosed with simple perimembranous VSD by transthoracic echocardiography (TTE) before the operation, without any other congenital cardiac malformation, without a history of cardiovascular surgery and without other systemic diseases. The indications for transthoracic device closure were suitable for all patients in this study. According to the anesthetic method, the patients were divided into two groups: 36 patients in group S, who were given sufentanil and sevoflurane-based fast-track cardiac anesthesia, and 32 patients in group F, who were given fentanyl and sevoflurane-based cardiac anesthesia.

In group S, all patients routinely fasted before anesthesia. After the patients entered the operating room, we cleaned one side of the forehead and temporal skin, fixed the sensor patches and connected the patches to the BIS-VISTA monitor. After the lead test was passed, the BIS value target range was set at 40-60, while the monitor automatically generated and recorded the BIS value, electromyography and signal quality index (SQI) each second^[7,8]^. The electrocardiogram and blood oxygen saturation were also monitored at the same time. Inhalational anesthesia induction was performed with 2-4% sevoflurane and 4 L/min oxygen. An intravenous cannula was inserted on the dorsum of the hand, and physiological saline was infused. Then 0.15 mg/kg intravenous cisatracurium besylate and 1 µg/kg sufentanil were given. After reaching an adequate depth of anesthesia, endotracheal intubation and mechanical ventilation were performed. The pressure-controlled ventilation was used, and the end-tidal carbon dioxide partial pressure was monitored. Invasive blood pressure was monitored through the radial artery, and central venous pressure was monitored in the subclavian vein. Body temperature was monitored as the nasopharyngeal temperature and was kept at approximately 36.5ºC. Anesthesia was maintained with 2% sevoflurane (1-1.5 MAC) inhalation and a continuous sufentanil infusion at a rate of 1 µg/kg/h. During the procedure, the sevoflurane dose was adjusted depending on the depth of anesthesia to maintain the BIS value. In group F, the preoperative preparation process was the same as in group S. Anesthetic induction consisted of inhalational 2-4% sevoflurane, intravenous 0.15 mg/kg cisatracurium besylate and 10 µg/kg fentanyl, while maintenance anesthesia consisted of fentanyl boluses at approximately 5 µg/kg/h and 2% sevoflurane (1-1.5 MAC) inhalation.

The procedure was completed and guided by transesophageal echocardiography (TEE). After successful general anesthesia, we performed a small incision of approximately 2-3 cm in the lower end of the sternum to access the pericardial cavity. After surgical exposure of the right ventricular free wall, heparinization (1 mg/kg) was administered intravenously. Next, the optimal puncture site was selected under TEE guidance. Then, the right ventricular puncture was performed using a needle at this site. A guidewire was inserted, and the right ventricle-VSD-left ventricle transport track was established by TEE guidance. Next, the occluder was sent and released to close the VSD. After the operation, all patients were sent to the intensive care unit (ICU) for further monitoring and treatment.

Clinical data included age, sex, body weight, operation time, extubation time, postoperative ICU stay, hospitalization days, complications, pediatric anesthesia emergence delirium (PAED) scale score and face, legs, activity, cry, consolability (FLACC) scale score. BIS values were recorded at 5 time points: before anesthesia induction (T1); the moment of endotracheal intubation (T2); the beginning of skin incision (T3); 30 minutes after operation (T4); and the end of operation (T5). After patients came back to consciousness, nurses scored the postoperative agitation and analgesia condition of the patients every 5 minutes and regarded the highest score as the final score.

The PAED scale score was used to measure emergence delirium in children in the following five psychometric items: 1) the child made eye contact with the caregiver; 2) the child’s actions were purposeful; 3) the child was aware of his/her surroundings; 4) the child was restless; and 5) the child was inconsolable^[9,10]^. Each item was scored from 1 to 4 (with reverse scoring when applicable), and the scores were added to obtain a total scale score. The degree of emergence delirium varied directly with the total score. A PAED score ≥5 meant the presence of emergence delirium.

The FLACC scale was used to assess pediatric postoperative pain^[11,12]^. The FLACC scale consists of five domains (face, legs, activity, cry, and consolability) with scores of 0, 1 and 2 for each domain and a total score ranging from 0 to 10. A FLACC score ≥3 was considered unsatisfactory analgesia.

The data of this study were analyzed using SPSS 22.0. Continuous data are expressed as mean ± standard deviation, and counting data are represented by usage rate (%). The rates of the two groups were compared by the chi-square test, and the continuous data between groups were compared by the independent samples t-test. *P*<0.05 was defined as statistical significance.

## RESULTS

There was no significant difference in age, sex ratio, body weight, operation time or sevoflurane inhalation time between the two groups (*P*>0.05) (Table 1). BIS values were collected and compared at the 5 time points, and there was no significant difference (*P*>0.05) (Table 2). There was no severe anesthesia or drug-related complications, such as death, occluder displacement, new complete atrioventricular block, new aortic valve regurgitation, heart failure, liver failure, or acute kidney injury in either group. There were some minor complications listed in Table 3. Extubation time, ICU stay, hospital stay, PAED score, FLACC score and incidence of emergence delirium in group S were significantly lower than in group F (*P*<0.05) (Table 1).

**Table 1:** General information in both groups.

Items	Group S	Group F	*P*-value
Age (years)	4.5±1.5	4.2±1.5	*P*=0.489
Gender (male/female)	20/16	19/13	*P*=0.101
Weight (kg)	16.9±3.1	16.5±2.7	*P*=0.581
Operation time (min)	47.1±15.9	49.6±16.8	*P*=0.539
Sevoflurane inhalation time (min)	43.6±15.2	45.5±16.1	*P*=0.620
Extubation time (min)	19.4±1.8	28.2±3.2	P*<0.001*
PEAD score	3.6±1.7	6.4±3.4	*P*<0.001
FLACC score	1.2±0.6	2.1±0.5	*P*<0.001
ICU stay (h)	6.1±1.1	7.6±2.1	*P*<0.001
Hospital stay (day)	2.7±0.7	4.1±1.4	*P*<0.001
Emergence delirium (%)	5.6	34.4	*P*=0.003

**Table 2:** Comparison of the BIS value at each time point.

Time	Group S	Group F	*P*-value
T_1_	95.1±2.3	95.7±1.9	*P*=0.259
T_2_	46.0±2.3	46.9±3.1	*P*=0.176
T_3_	48.2±1.4	48.9±2.1	*P*=0.091
T_4_	51.2±1.8	52.2±2.2	*P*=0.056
T_5_	55.9±1.8	57.4±3.1	*P*=0.063

**Table 3:** Comparison of minor complications in both groups.

Adverse reaction	Group S	Group F	*P*-value
Nausea and vomiting (%)	11.1	18.8	*P*=0.375
Drowsiness (%)	13.9	18.8	*P*=0.587
Dizziness (%)	2.8	6.3	*P*=0.681

## DISCUSSION

VSD is one of the most common congenital cardiac malformations^[13]^. Both transcatheter device closure and traditional surgical repair under cardiopulmonary bypass have had definite success and have been widely applied. In recent years, transthoracic device closure of VSD has gradually become an alternative treatment for children with VSD. The advantages of this method are that it is easy to establish the delivery set, it is easy to operate and learn, the incision is small and cosmetic, and the recovery is quick after operation^[2,14,15]^. Our cardiac center reported a larger series of cases from our experience with this technique^[2]^. Fast-track cardiac anesthesia for children undergoing transthoracic device closure of VSD has been carried out in our center in an attempt to help patients recover faster by shortening the anesthesia time, hastening the removal of the endotracheal tube and reducing the ICU stay^[16,17]^. Emergence delirium on awakening from general anesthesia would definitely affect postoperative recovery.

Sevoflurane is generally the preferred anesthetic agent to induce and maintain cardiac general anesthesia in children due to its rapid induction and recovery characteristics, easily controlled anesthetic depth, stable hemodynamics and minor effect on cardiac function^[18]^. However, in recent years, it has been shown that one of the main complications after sevoflurane anesthesia in children is emergence delirium upon awakening from general anesthesia, with a reported incidence ranging from 10% to 80%, especially in preschoolers^[3,19]^. This agitation has a variety of manifestations, including crying, excitement and delirium after awakening. Although emergence delirium is self-limited, it generally does not lead to permanent sequelae. Patients with persistent severe postoperative delirium have negative implications and experience less satisfaction, as do their families, due to the potential for self-injury and other injurious behaviors, being a burden on nursing staff. It has been shown that low-dose fentanyl (an induced dose of less than 1 µg/kg) used in children after sevoflurane-based anesthesia can reduce the incidence of emergence delirium without prolonged hospital stay^[20,21]^. Sufentanil, as a derivative of fentanyl, is a lipophilic opioid agonist that can easily pass through the blood-brain barrier and bind to plasma proteins at a higher rate than fentanyl^[22]^. It has high selectivity for opioid receptors, leading to analgesia and sedation. Clinical studies have shown that sufentanil has the highest therapeutic index of all opioids^[23]^. As a result, sufentanil can provide stronger analgesic effects over a longer period than the same dose of fentanyl or morphine and can reduce respiratory depression and the incidence of adverse reactions^[6,23]^. Li and his team reported that administration of sufentanil could reduce emergence agitation in children receiving sevoflurane anesthesia for adenotonsillectomy compared to fentanyl, without delaying the recovery time or causing significant hypotension^[24]^. Luo et al. found that dexmedetomidine combined with sufentanil was able to effectively decrease the incidence of emergence agitation in children receiving sevoflurane anesthesia for cleft palate repair surgery^[25]^. Liang et al. concluded that sufentanil is better than fentanyl in reducing emergence agitation in children anesthesized with sevoflurane in their study^[26]^.

In this study, there were no statistically significant differences in age, sex, weight, operation time or sevoflurane inhalation time between the two groups. The mean arterial pressure, heart rate and blood oxygen saturation of the two groups were good throughout the monitoring process. There was no significant difference in BIS between groups at each time point. The safety of the clinical application was the same. Since the operation time of transthoracic device closure of VSD in children is short, there is no need for long-term sedation, analgesia or anesthesia. In the premise of obtaining the same anesthetic effect, compared to group F, patients in group S who received sufentanil during anesthesia maintenance had a lower incidence of emergence delirium and a lower FLACC score. The results showed that the effect of sevoflurane combined with intravenous sufentanil pumping was better than the effect of sevoflurane inhalation combined with fentanyl, which was beneficial for postoperative recovery and perioperative management of patients. This was due to the powerful analgesic effect of sufentanil, which enhances the sedative effect of sevoflurane and reduces the side effects of delirium.

Because sufentanil has dose-dependent respiratory inhibition, it is necessary to pay attention to its dosage during use^[22]^. When combined with central nervous system inhibitors, sufentanil may have additional pharmacological effects that increase the likelihood of adverse reactions, such as respiratory depression. If it is inevitable to use them at the same time, the dose and duration of sufentanil treatment should be reduced (to the minimum requirement), and the hemodynamics and respiratory status of patients should be closely monitored. Sufentanil use should be carefully monitored in patients at risk of intestinal obstruction or in patients with existing intestinal obstruction or biliary tract disease^[22]^. In this study, there were no significant adverse reactions in the sufentanil group, which confirmed the safety of the regimen.

There were also some shortcomings to this study. It was a retrospective analysis, not a prospective, randomized, controlled, double-blind study, and there was a deviation in the selection of cases, but the results supported the conclusion to some extent. We look forward to future prospective, double-blind, controlled trials, as well as multicenter studies to determine whether our conclusions are correct and feasible.

## CONCLUSION

Continuous intravenous infusion of sufentanil can be safely used in children undergoing transthoracic device closure of VSD under fast-track cardiac anesthesia with sevoflurane. Compared to fentanyl, sufentanil is more effective in reducing the incidence of emergence delirium, obtaining a lower postoperative analgesic score and higher comfort. It is beneficial to the recovery of patients after operation and it is worth popularizing.

**Table:** 

Authors' roles & responsibilities	
NX	Substantial contributions to the conception or design of the work; or the acquisition, analysis or interpretation of data for the work; drafting the work or revising it critically for important intellectual content; final approval of the version to be published.
QC	Substantial contributions to the conception or design of the work; or the acquisition, analysis or interpretation of data for the work; drafting the work or revising it critically for important intellectual content; final approval of the version to be published.
STH	Acquisition, analysis or interpretation of data for the work; final approval of the version to be published.
KPS	Acquisition, analysis or interpretation of data for the work; final approval of the version to be published.
HC	Substantial contributions to the conception or design of the work; or the acquisition, analysis or interpretation of data for the work; drafting the work or revising it critically for important intellectual content; final approval of the version to be published.
NX	Substantial contributions to the conception or design of the work; or the acquisition, analysis or interpretation of data for the work; drafting the work or revising it critically for important intellectual content; final approval of the version to be published.
QC	Substantial contributions to the conception or design of the work; or the acquisition, analysis or interpretation of data for the work; drafting the work or revising it critically for important intellectual content; final approval of the version to be published.
STH	Acquisition, analysis or interpretation of data for the work; final approval of the version to be published.
KPS	Acquisition, analysis or interpretation of data for the work; final approval of the version to be published.
HC	Substantial contributions to the conception or design of the work; or the acquisition, analysis or interpretation of data for the work; drafting the work or revising it critically for important intellectual content; final approval of the version to be published.
